# Microscopic and submicroscopic exploration of diplolepideae peristome structures in hygroscopic movement

**DOI:** 10.1186/s12870-024-05407-8

**Published:** 2024-07-26

**Authors:** Yanzhi Wu, Qimei Wu, Zhaohui Zhang, Zhihui Wang

**Affiliations:** 1 School of Life Sciences, Guizhou Normal University, Guiyang, 550025 China; 2 Key Laboratory for Information System of Mountainous Area and Protection of Ecological Environment of Guizhou Province, Guizhou Normal University, Guiyang, 550025 China

**Keywords:** Mosses, Diplolepideae, Arthrodontae, Peristomes, Microfibrils, Mechanism of hygroscopic movement

## Abstract

**Background:**

The Diplolepideae are the larger group within the Arthrodontae mosses, characterized by peristomes formed from residual cell walls. It is now understood that these peristomes exhibit diverse hygroscopic movements, playing a crucial role in spore release. However, the exact mechanism behind this movement remains unclear, lacking direct evidence. This study investigated the microscopic and submicroscopic structures of the peristomes in three Diplolepideae species: *Hypopterygium fauriei* (Besch.), *Pylaisia levieri* (Müll. Hal.) Arikawa and *Regmatodon declinatus* (Hook.) Brid. Scanning electron microscopy (SEM) and transmission electron microscopy (TEM) were used to reveal the differences in their hygroscopic movement mechanisms.

**Results:**

The three species exhibited distinct responses upon wetting: *H. fauriei*’s exostome closed inwards, *P. levieri*’ opened outwards, and *R. declinatus*’ elongated significantly. These differences are attributed to the varying microfibril deposition in the exostome layers. Uniform deposition in the inner layer and minimal deposition in the outer layer enabled exostome opening upon wetting and closing when dry. Our findings suggest that the diastole and contraction of fine microfibrils in the exostome plates and ridges are the key drivers of hygroscopic movement.

**Conclusions:**

This study provides further evidence at both the structural and submicroscopic levels, contributing to the unraveling of the hygroscopic movement mechanism in Diplolepideae peristomes. This enhanced understanding sheds light on the relationship between peristome structure and function.

**Supplementary Information:**

The online version contains supplementary material available at 10.1186/s12870-024-05407-8.

## Introduction

Hedwig first reported on peristomes in the eighteenth century [1]. Peristomes, vital structures located at the top of moss capsules, are classified into two types based on their development: nematodontae and arthrodontae [2, 3]. Nematodontae peristomes develop from multiple layers of intact cells, while arthrodontae form through the fusion of amphithecium cell walls [4]. Within the arthrodontae group, further classification divides them into haplolepideae and diplolepideae based on the specific amphithecium cell walls that contribute to their residual cell walls [5]. Diplolepideae peristomes possess both endostomes and exostomes. The exostomes consist of structures like plates, middle and transverse raphes on their lateral aspect, and medial structures such as transverse and longitudinal lamellae. Endostomes, on the other hand, are simpler in structure, containing components like tubercles and tresis [6–8]. This difference in complexity likely arises from the uneven deposition of fibers in the exostomes compared to the lesser deposition in the endostomes [3, 9]. The primary components of the diplolepideae peristome structure are cellulose and pectin. Notably, the type and content of these components vary depending on both the cell type and the stage of development of the moss plant [10, 11].

Currently reported, such as the genus *Timmia* exhibit hygroscopic movement. This movement involves a downward pressurization of the middle and upper portions of the capsule teeth. The apical (tip) region curves inwards, skimming along the dome-shaped endostomes. Ultimately, the exostome tips join pairwise, forming eight triangular points. These points possess broad and obtuse apices, rendering them incapable of penetrating between the endostomes [12]. In contrast, the transition from wet to dry state in Brachytheciaceae is characterized by a rapid outward oscillation of the outer teeth [8]. The movement of *Brachythecium velutinum* Schimp. peristomes is similar to that of *Timmia megapolitana* but differs during drying. In this respect, the exostomes open briefly in the middle of the teeth’s upper part before extending inside the endostomes [13]. Besides, the exostomes of *Bryocrumia vivicolor* Buck. are inwardly curved when dry [14]. The tips of *Funaria hygrometrica* peristome exostomes curve inwards during hygroscopic movement, passing through the interstices between the endostomes. Conversely, they curve outwards during drying. The denticles on the dorsal side of the exostomes may temporarily adhere to spores [8]. In *Bryum*, the exostomes are bent downwards when dry, with their tips inserted into the gaps between the endostomes [15]. A well-developed bilayer structure in capsule teeth could assist in hindering spore release. This is supported by the observation that some moss peristomes, like *Terophyllium nemorosum*, break all their bilayer teeth after undergoing repeated (10–20 times) hygroscopic cycles [16]. Notably, there is scarce information on hygroscopic movement of peristomes in the open state, during both wet and intermediate conditions [17].

Currently, direct evidence for the mechanism of peristome movement in Diplolepideae remains elusive. The factors driving the variability in their movement directionality and swelling at different sites are unclear. The variability in curvature direction, site, and sensitivity across different peristomes remains a topic of debate. While Steinbrinck attributed the movement to capsule expansion and structural arrangement [17], Schnepf et al. demonstrated that the motion of the exostomes of moss is driven by different expansion rates of the outer layers (“plates”) and inner layers (“ridges”), and suggested that the diversity is caused by two substances, suberin-like substances and wax-lamellae [18]. Steinbrinck proposed that the accumulation of solid particles within the capsule caused its volume to increase during water absorption, triggering hygroscopic movement. He further attributed the differences in movement between different peristome parts to their structural arrangement [17]. It has been suggested that the movement of exostomes, the outer layer of the peristome, might be related to the thickening of specialized walls within the structure. Additionally, the presence of hydrophobic materials on the exostome surface was proposed to completely seal it, restricting water flow and delaying expansion and contraction, thus impacting the movement [5, 18]. In addition, some have shown that the bending movement of the exostomes was mainly driven by the outer layer of the outer tooth, or by the change of the microfibril orientation [13, 19, 20].

A key challenge in understanding moss peristome movement is the lack of direct evidence for the underlying cause of their directional and variable expansion at different sites. We hypothesize that this variability is linked to the specific structural features and internal fiber arrangements within the peristome. To investigate this, we selected three distinct types of exostome hygroscopic movement with significantly different capsule tooth morphologies: *Hypopterygium fauriei*, *Pylaisia levieri*, and *Regmatodon declinatus* (See Table 1 for abbreviations of relevant terms). Through scanning electron microscopy (SEM) and transmission electron microscopy (TEM) observations and analyses, this study aimed to reveal the microscopic and submicroscopic structural features of the peristomes associated with these three distinct hygroscopic movement types. Ultimately, this work contributes to a deeper understanding of how peristomes respond to environmental changes.

**Table 1:** Summary of study species

Family	Species	Location sites	Habitat
Hypopterygiaceae	*Hypopterygium fauriei* (Besch.)	Karst Sinkhole in Kaiyang, Guizhou province, China; E107°00′12″, N27°05′03″	Growing on wet rocks, 1058 ± 3 m.
Hypnaceae	*Pylaisia levieri* (Müll. Hal.)	Karst Sinkhole in Kaiyang, Guizhou province, China; E106°50′70″, N27°08′20″	Growing on exposed trunks, 1065 ± 3 m.
Leskeaceae	*Regmatodon declinatus* (Hook.) Brid.	Xinyuan, Maojian Town, Duyun City, Guizhou Province, China ; E107°26′85″, N26°14′41″	Growing on exposed trunks, 1133 ± 2.5 m.

## Materials and methods

### Research species

We chose three mosses, *Hypopterygium fauriei* [21], *Pylaisia levieri* [22] and *Regmatodon declinatus* [21], as our study material, and their capsule morphology differed significantly (Table 1).

### Optical microscope observation

To study the detailed structure of the capsules, morphological intact capsules were selected from dried capsules. The sporangia were carefully air-dried at room temperature for 24 h to preserve their delicate structures. An OLYMPUS CX41 microscope combined with Image View software was used to obtain the capsule images. After the dry state capsule picture was obtained, it was submerged in water and humidified for 20 min. Finally, the excess water was wiped off and pictures were captured again. This observation process was repeated for a total of three spore capsules.

### Scanning electron microscopy (SEM, Servicebio, Wuhan, China)

Freshly collected (*n* = 10) capsules were first placed in 10 ml PE centrifuge tubes containing 70% ethanol FAA fixative for preservation and transport at 4 °C. Upon arrival at the lab, they underwent a series of treatments for SEM analysis: (1) fixation with 1% OsO_4_ for 1–2 h at room temperature, (2) dehydration through increasing concentrations of ethanol and isoamyl acetate, (3) drying with a Critical Point Dryer (Quorum K850), (4) conductive metal coating by attaching them to stubs and sputter-coating with gold for 30 s, and finally, (5) observation and imaging using a Hitachi SU8100 scanning electron microscope.

### Transmission electron microscopy (TEM, Servicebio, Wuhan, China)

Fresh spore capsules (*n* = 10) collected in the field were immediately preserved and transported in 0.5 ml PE centrifuge tubes containing 2.5% glutaraldehyde fixative (pH 7.0-7.5) at 4 °C. Upon arrival at the laboratory, the samples underwent the following processing steps: (1) Fixation with 1% OsO_4_ for 7 h at room temperature; (2) Dehydration through a graded series of ethanol solutions; (3) Resin infiltration with EMBed 812 at increasing concentrations and incubation at 37 °C; (4) Polymerization of the resin blocks containing capsules in a 65 °C oven for over 48 h; (5) Ultra-thin sectioning of the resin blocks at 60–80 nm using a Leica UC7 ultramicrotome, followed by retrieval of the sections onto 150 mesh copper grids coated with formvar film; (6) Staining with 2% uranyl acetate solution for 8 min and 2.6% lead citrate solution for 8 min (both avoiding light and CO_2_ exposure), followed by rinsing with ethanol and ultrapure water; and (7) Observation and image capture using a Hitachi HT7800 transmission electron microscope.

## Results

### Hygroscopic movement of three mosses

**Fig. 1:**
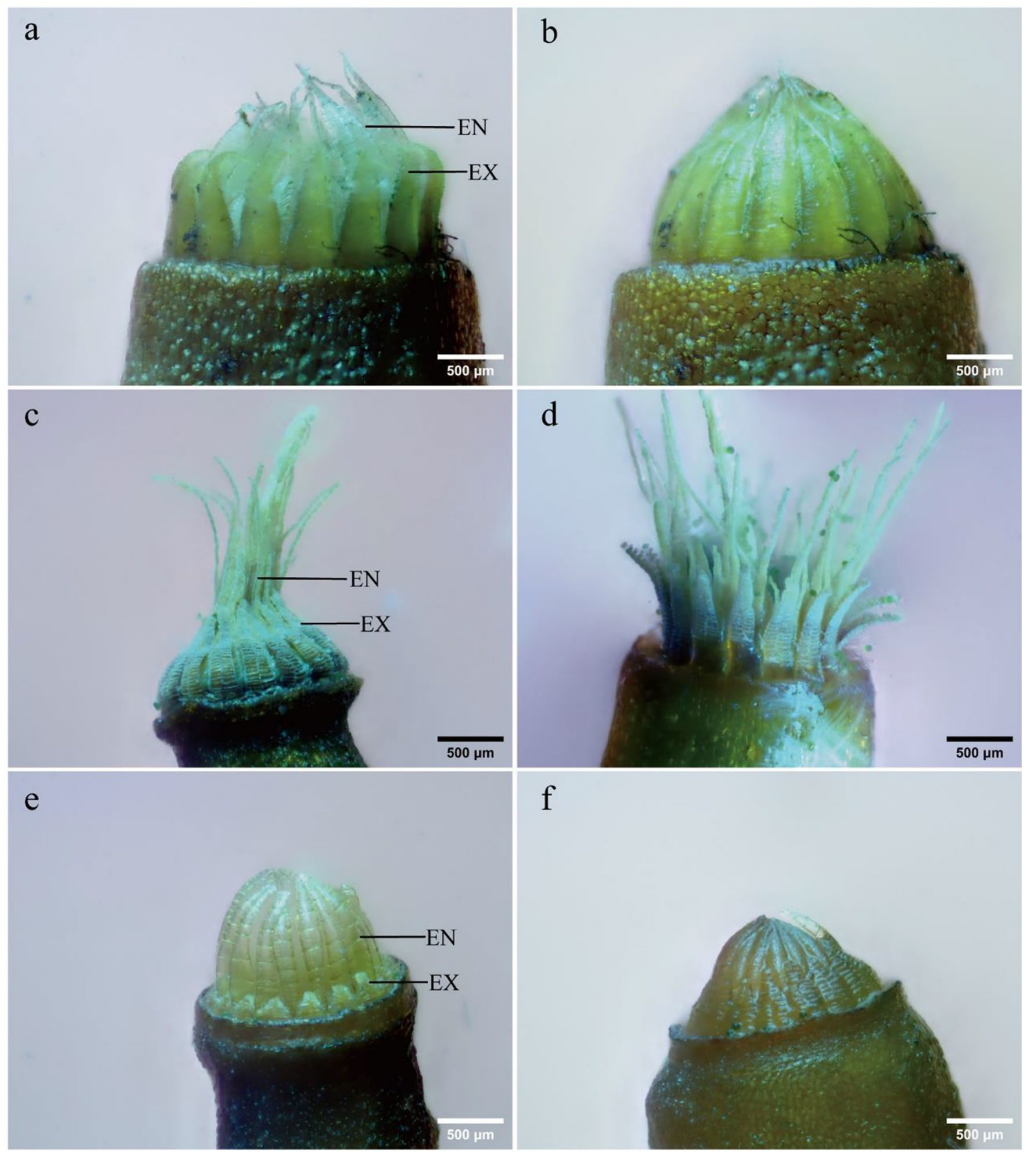
Peristome hygroscopic movement (**EN**: endostomes; **EX**: exostomes). **a-b**: *H. fauriei*; **a**: Dry state, **b**: Wet state; **c-d**: *P. levieri*; **c**: Dry state, **d**: Wet state; **e-f**: *R. declinatus*; **e**: Dry state, **f**: Wet state; (For dynamic processes, see Support information videos 1–3.)

Upon exposure to humidity, the three moss species exhibited distinct peristome movements. In *H. fauriei*, the middle parts of the exostomes closed inwards, while the endostomes remained unchanged (Fig. 1a, b). *P. levieri* displayed outward opening of the exostomes and remained open endostomes (Fig. 1c, d). *R. declinatus*, however, presented a unique “telescopic” movement where the entire exostome segment contracted and straightened, significantly narrowing the endostome gap (Fig. 1e, f). These observations categorize *H. fauriei* and *P. levieri* as “tension-type” due to their obvious inward/outward bending, while R. *declinatus* belongs to the distinct “telescopic” type with no bending [23].

### The structural features of the three mosses observed by SEM

#### *H. Fauriei*

**Fig. 2:**
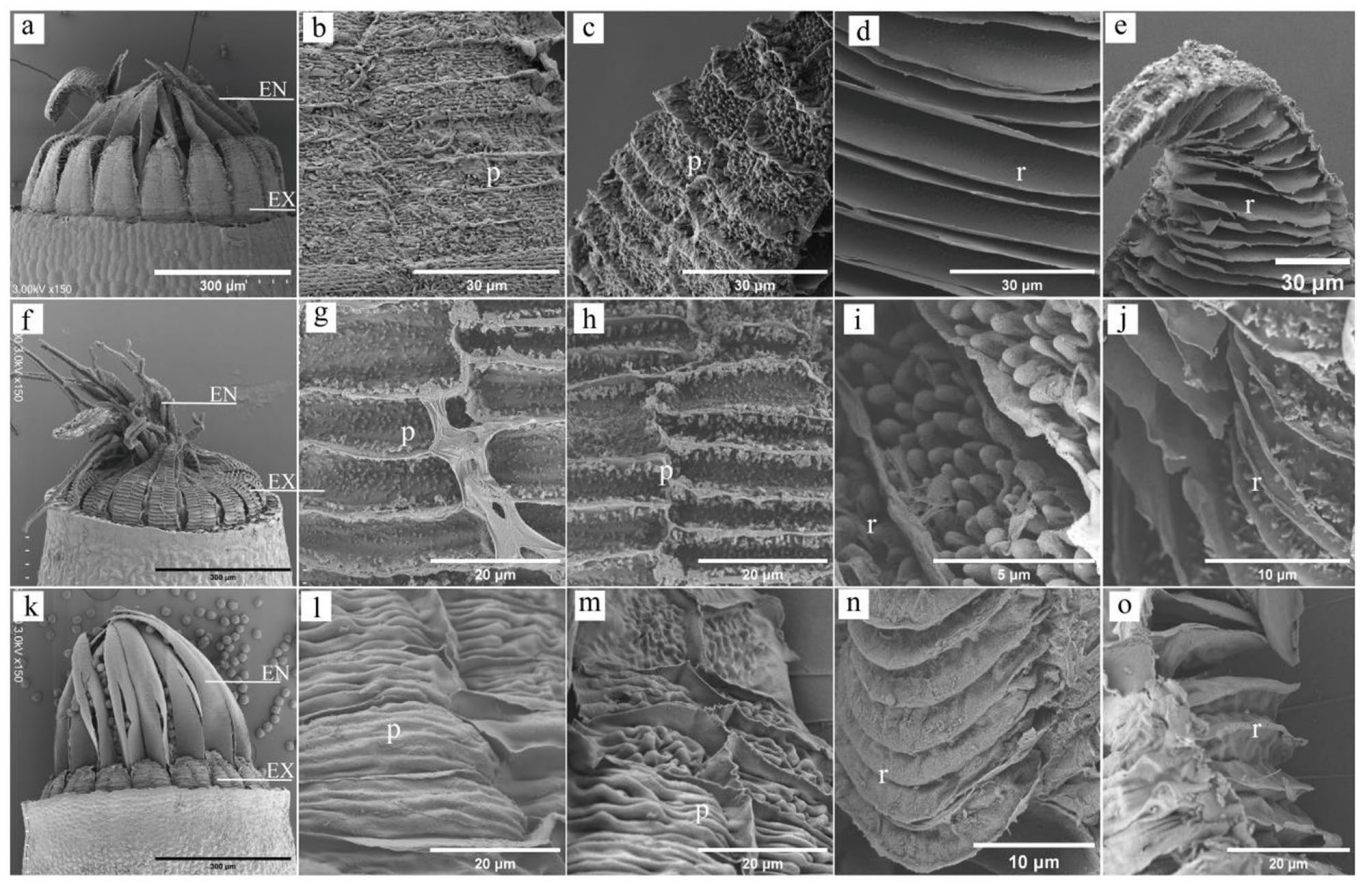
SEM image of peristomes (**EN**: endostomes; **EX**: exostomes; **p**: outer plates; **r**: inner ridges). **a-e**: *H. fauriei*; **a**: Peristomes, **b**: The base of exostomes dorsal surface; **c**: The middle of exostomes dorsal surface, **d**: The base of exostomes ventral surface, **e**: The middle of exostomes ventral surface; **f-j**: *P. levieri*; **f**: Peristomes, **g**: The base of exostomes dorsal surface, h. The middle of exostomes dorsal surface, **i**: The base of exostomes ventral surface, **j**: The middle of exostomes ventral surface; **k-o**: *R. declinatus*; **k**: Peristomes, **l**: The base of exostomes dorsal surface, **m**: The middle of exostomes dorsal surface, **n**: The base of exostomes ventral surface, **o**: The middle and side of the exostomes cusp

**Fig. 3:**
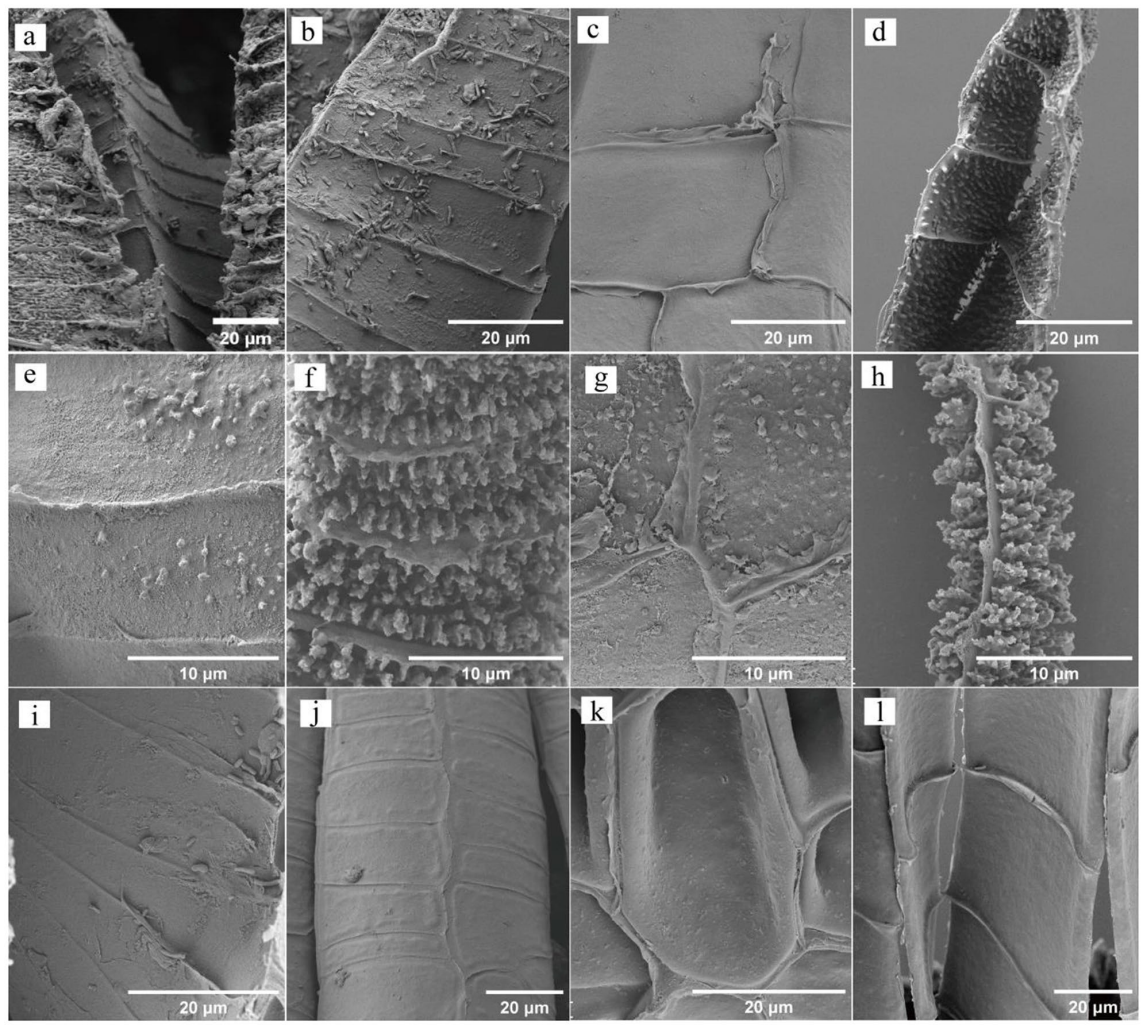
SEM image of the endostomes. **a-d**: *H. fauriei*; **a**: The base of dorsal surface, **b**: The medial of dorsal surface, **c**: The base of ventral surface, **d**: The middle of ventral surface; **e-h**: *P. levieri*; **e**: The base of dorsal surface, **f**: The medial of dorsal surface, **g**: The base of ventral surface, **h**: The middle of ventral surface; **i-l**: *R. declinatus*; **i**: The base of dorsal surface, **j**: The medial of dorsal surface, **k**: The base of ventral surface, **l**: The middle of ventral surface

The dorsal surface of its exostomes lacks concavity and exhibits transverse stripes at the base. The middle section appears slightly dented and adorned with papillae (Fig. 2a, b and d). Conversely, the ventral surface’s base boasts a flattened, smooth, and lamellate structure, transitioning to a narrowly flattened and mastoid middle section (Fig. 2a, c and e). Its endostomes display smooth lateral surfaces on the dorsal side, while the ventral surface features a smooth base and a middle section adorned with coarse mastoid protrusions (Fig. 3a-d).

#### *P. Levieri*

This species showcases a significantly different exostome structure. The dorsal surface base dips inwards noticeably and bears fine warts (Fig. 2f, g and i). The middle parts exhibit a marked depression and are decorated with dendritic warts. The ventral surface’s base extends long and flat, adorned with flaky and coarse warts, while the middle section remains flat and flaky but features finer warts (Fig. 2f, h and j). Similarly, the endostomes present a fine wart on the dorsal surface base and a dendritic wart in the middle. Interestingly, the ventral surface structure mirrors the dorsal one (Fig. 3e-h).

#### *R. Declinatus*

Unlike the previous species, its exostomes lack a sunken base on the dorsal side, displaying wrinkles instead. The middle section shows a slight depression and bears warts (Fig. 2k, l and n). Remarkably, both the medial base and middle parts of the ventral surface exhibit a smooth, flat triangular shape (Fig. 2k, m and o). Both sides of the endostomes remain smooth across the entire structure (Fig. 3i-l).

### The structural features of the three mosses observed by TEM

**Fig. 4:**
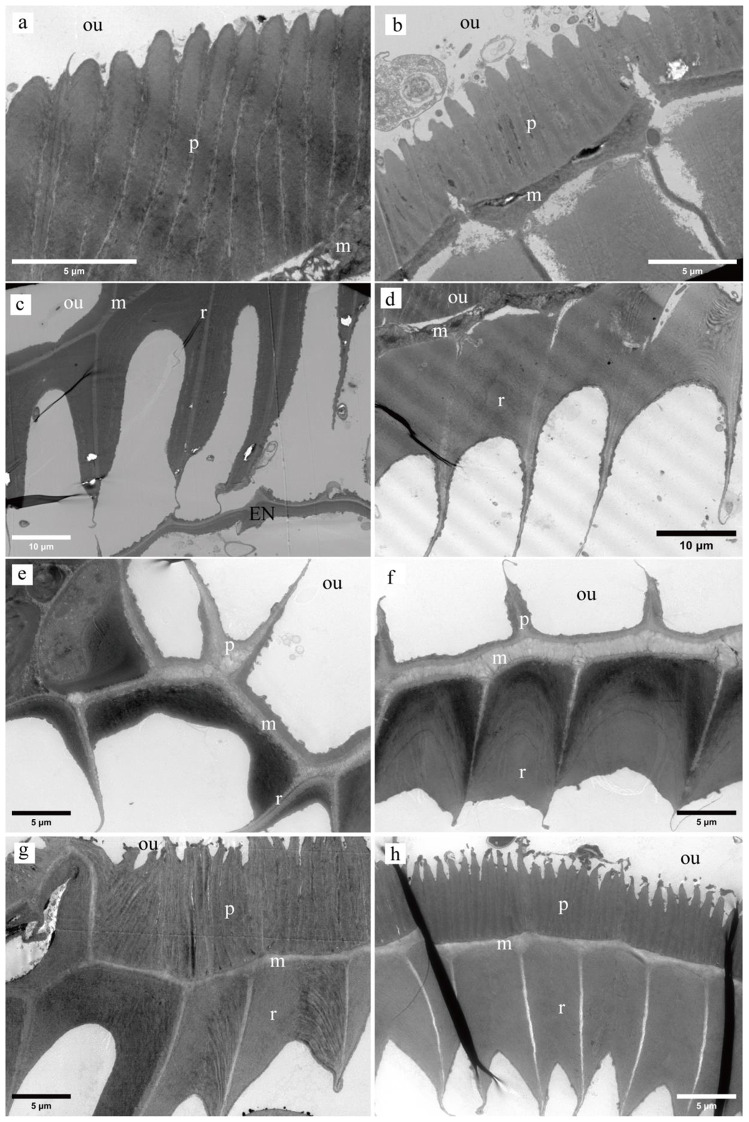
TEM image of the exostomes (**p**: outer plates; **r**: inner ridges; **m**: middle layer; **ou**: outside). **a-d**: *H. fauriei*; **a**: The base of plates, **b**: The middle of plates, **c**: The basal of ridges, **d**: The middle of ridges; **e-f**: The plates and ridges of *P. levieri*; **e**: Base, **f**: middle; **g-h**: The plates and ridges of *R. declinatus*; **g**: Base, **h**: middle

Microscopic analysis revealed distinct differences in fiber arrangement and thickness between the three moss species studied. In *H. fauriei*, the exostome outer plates displayed compactly arranged fibers at the base and sparse fibers in the middle (Fig. 4a, b). The inner ridge fibers were thin at the base with a tapered tip, transitioning to thick and sharp-tipped fibers in the middle (Fig. 4c, d). A clear middle layer composed of fibers separated the outer plates and inner ridges (Fig. 4a-d).

*P. levieri* exhibited very few fibers in the base and middle of its exostome outer plates. Similar to *H. fauriei*, the inner ridge fibers were thin and tapered at the base, thickening with blunt tips in the middle (Fig. 4e, f). Notably, the middle layer in *P. levieri* was significantly wider than in *H. fauriei*.

*R. declinatus* shared some similarities with *H. fauriei* in terms of fiber arrangement. Its exostome outer plates also had compact fibers at the base and sparser fibers in the middle (Fig. 4g, h). The inner ridge fibers followed the same pattern of thin base with a tapered tip and thick, sharp-tipped middle section. Interestingly, the middle layer in *R. declinatus* resembled that of *H. fauriei* in structure.

In contrast to the exostomes, the endostomes of all three moss species displayed a monolayer structure devoid of plates and ridges, with fibers arranged in a single layer (Figure S1b, d, f).

### The fiber arrangement of the three mosses observed by TEM

**Fig. 5:**
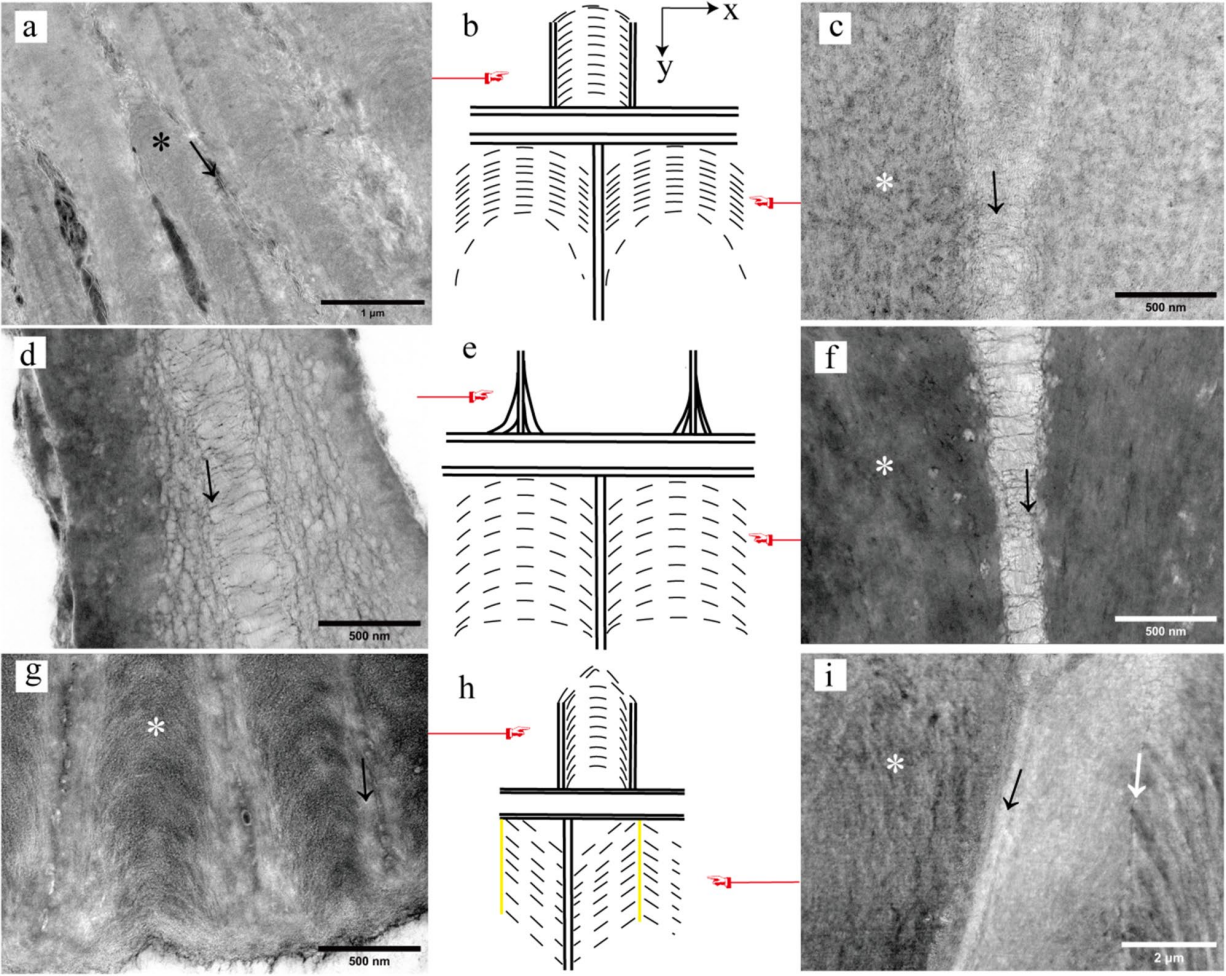
Arrangement of microfibrils on the exostomes (black arrow indicates the concentrated distribution area of coarse microfibrils; The asterisk indicates the concentrated distribution area of fine microfibrils; The white arrow is the fiber dividing line at the inner ridges; Because the microfibrils of the outer plates and inner ridges at the base and middle are arranged in the same way, we only select the pictures at the base for display). **a-c**: *H. fauriei*; **a**: The plates, **b**: The microfibrils arrangement diagram (Dotted lines represent fine microfibrils; Solid lines represent coarse microfibrils), **c**: The ridges; **d-f**: *P. levieri*; **d**: The plates, **e**: The microfibrils arrangement diagram, **f**: The ridges; **g-i**: *R. declinatus*; **g**: The plates, **h**: The microfibrils arrangement diagram (solid yellow lines indicate fiber boundaries), **i**: The ridges

The exostomes of the investigated moss species (*H. fauriei*, *R. declinatus*, and *P. levieri*) exhibit differences the microfibril composition of their outer plates and inner ridges, as illustrated in Fig. 5. Notably, both fine and coarse microfibrils are present on the outer plates of *H. fauriei* and *R. declinatus*, indicating substantial fiber deposition in these species. However, *P. levieri* displays significantly less fiber deposition on its outer plates, with only coarse microfibrils being detectable (Figs. 4a, b and e-h and 5a, b, d, g and h). Interestingly, all three moss species possess both fine and coarse microfibrils on their inner ridges (Fig. 4b, c, e, f, h, and i).

In the outer plates of *H. fauriei* exostomes, fine microfibrils display an axial, arc-like arrangement, contrasting with the radial orientation of the coarser microfibrils (Fig. 5a and b). This pattern persists in the inner ridges, where the fine microfibrils maintain their axial, arc-like alignment, while the coarse microfibrils remain radially oriented (Fig. 5b and c). Notably, the middle layer presents a chaotic and dense microfibrillar network, predominantly composed of radially and axially arranged coarse microfibrils (Figure S1a).

Axial fine microfibrils and axial coarse microfibrils are also present in the endostomes of the three mosses (Figure. S1b, d and f).

The exostomes of *P. levieri* exhibit distinct microfibril arrangements in different layers. As depicted in Fig. 4e, f, and 5d, e, the outer plates feature radially arranged coarse microfibrils. In contrast, the inner ridges display a combination of arrangements: fine microfibrils follow an axial, arc-like pattern, while coarse microfibrils maintain a radial orientation (Fig. 5e, f). The middle layer, visualized in Figure S1c, exhibits a simpler and looser arrangement, primarily consisting of axially oriented coarse microfibrils.

The outer plates of *R. declinatus* exostomes exhibit a distinct organization of microfibrils. Fine microfibrils are disposed in an axial arcuate pattern, contrasting with the (Fig. 5e and f) radial arrangement of coarserness microfibrils (Fig. 5g and h). This organization persists within the inner ridges, where fine microfibrils maintain their axial arcuate alignment, while coarser microfibrils remain radially disposed. Notably, the fine microfibrils within the depression are uniquely configured at a 45° angle on either side, forming a distinct fibrous demarcation line (Fig. 5h and i). The middle layer displays a less organized and looser microfibril architecture, primarily consisting of axial coarse microfibrils (Figure S1e).

## Discussion

The morphology and structure of diplolepideae peristomes are known to influence hygroscopic movement.

Previous studies on moss peristome function have shown that spore release primarily occurs through two mechanisms: destructive movement and persistent hygroscopic movement of the capsule teeth [12]. Peristomes, a defining feature of most mosses, consist of two concentric rings of teeth exposed after the calyptra detaches. These exposed peristomes undergo repeated cycles of hygroscopic movement in response to changes in ambient humidity, facilitating enhanced spore release [24]. The intricate structure of moss peristomes, responsible for spore dispersal, gives rise to a fascinating diversity of hygroscopic movements observed across various moss groups [3, 25, 26]. Steinbrinck’s [17] pioneering work dissected the intricate relationship between peristome structure and hygroscopic movement in mosses. He identified three types of motions based on exostome behavior: inward arching (dominated by longitudinal dorsal and transverse ventral structures), outward arching (dominated by the opposite configuration), and contraction-expansion with oscillation (obliquely striated dorsal surface). Patterson and Smith explored the opening motion in the wet state, suggesting its potential role in spore dispersal [16]. Diplolepideae peristomes exhibit a high degree of variation in both morphology and structure, which is reflected in the diversity of their hygroscopic movements. We have further categorized these hygroscopic movements into three distinct types: ‘wet-closed’, ‘wet-open’ and ‘wet-elongated’. Different hygroscopic movement types have different ecological significance Hygroscopic movement of the capsule teeth during drying plays a crucial role in spore release [8]. When air humidity is low, the exostomes open, the spores are released until these conditions are met [12, 27]. This process is hindered by closed teeth, which remain clamped shut under these conditions [28]. During drying, the bent exostomes vibrate in response to wind, facilitating the release of dry, powdery spores from the capsule interior. While closed peristomes can exhibit some movement during drying, they only open to form spore release channels at high air humidity. Some scholars believe that aerial dispersal in humid air might be preferable to dispersal in dry air [8, 12, 29]. This strategy potentially extends spore survival time, with spore release peaking after a period of high humidity. Additionally, it allows for spore release throughout a prolonged drying process, helping spores avoid unfavorable environments. An example of this strategy is the sub-hygroscopic movement observed in *Polytrichaceae* mosses, which promotes spore release during increased humidity and is believed to favor post-release spore germination [8, 12, 29, 30]. Conversely, hygroscopic movement in an intermediate state is associated with poor spore release, as the spores are not effectively dispersed in either dry or wet environments [23]. In conclusion, the state of hygroscopic movement is a critical factor for spore release in mosses. The impact of this movement on spore dispersal varies significantly across different moss species.

### Influence of exostome plates and ridges on hygroscopic movement

The outer plates and inner ridges of exostomes influence the direction and degree of hygroscopic movement, with varying effects on different parts. While fiber deposition on the inner ridges exhibits little variation, significant differences occur on the outer plates. When the amount of fiber deposition is similar on both plates and ridges, the hygroscopic movement and tension properties of the exostomes are minimal. Conversely, when the fiber deposition differs significantly, these properties become pronounced (Figs. 1, 2 and 4). Exostomes of *H. fauriei* and *R. declinatus* display more fiber deposits on the plates, resulting in a fuller dorsal surface. In contrast, the inner ridge fibers are deposited evenly. When both outer and inner fibers expand, the exostomes close (Fig. 2a-c). In contrast, *P. levieri* exhibits minimal fiber deposition on its outer plates, forming deep grooves on the surface. The inner layer ridge fibers are evenly deposited and structurally full. When the inner fibers become wet, they expand, causing the exostomes to open (Figs. 2d-f and 4e and f) [22].

The degrees of motion observed in different parts of exostomes are primarily determined by the layering of the outer plates and inner ridges, as well as their capacity for expansion and contraction under dry and wet conditions (Figs. 2 and 4) [17]. In *H. fauriei*, the fiber deposits on the middle part of the outer plates are less than those at the base, while the inner ridges are thicker and more uniform. This leads to a larger expansion volume of the inner layer compared to the outer layer in the middle plate under wet conditions, resulting in greater movement in the middle plates (Figs. 1a and b and 2a-e, and Fig. 4a-d). In contrast, *P. levieri* has very few fiber deposits on the outer plates. Consequently, the movement degree of its exostome parts is mainly controlled by the inner ridge fibers, with less fiber deposition at the base and more in the middle. Under wet conditions, the expansion volume of the inner layer is larger than that of the outer layer, leading to greater movement in the middle and upper parts (Figs. 1c and d, 2f-j and 4e and f). For *R. declinatus*, the deposition of the outer plates resembles that of the inner ridges. This results in similar expansion volumes of the inner and outer layers in the base and middle parts under wet conditions, leading to no significant difference in movement between the parts (Figs. 1e and f, 2k-o and 4g and h) [23]. This variation in exostome structure stems from the uneven deposition of cellulose during their development (Fig. 2) [4].

The endostomes lack both plates and ridges, consequently exhibiting no hygroscopic movement. This translates to no difference in bending direction or degree of movement across different parts during the process (Fig. 3 and Figure S1b, d, f).

### Microfibrils as the driving force of exostome movement

**Fig. 6:**
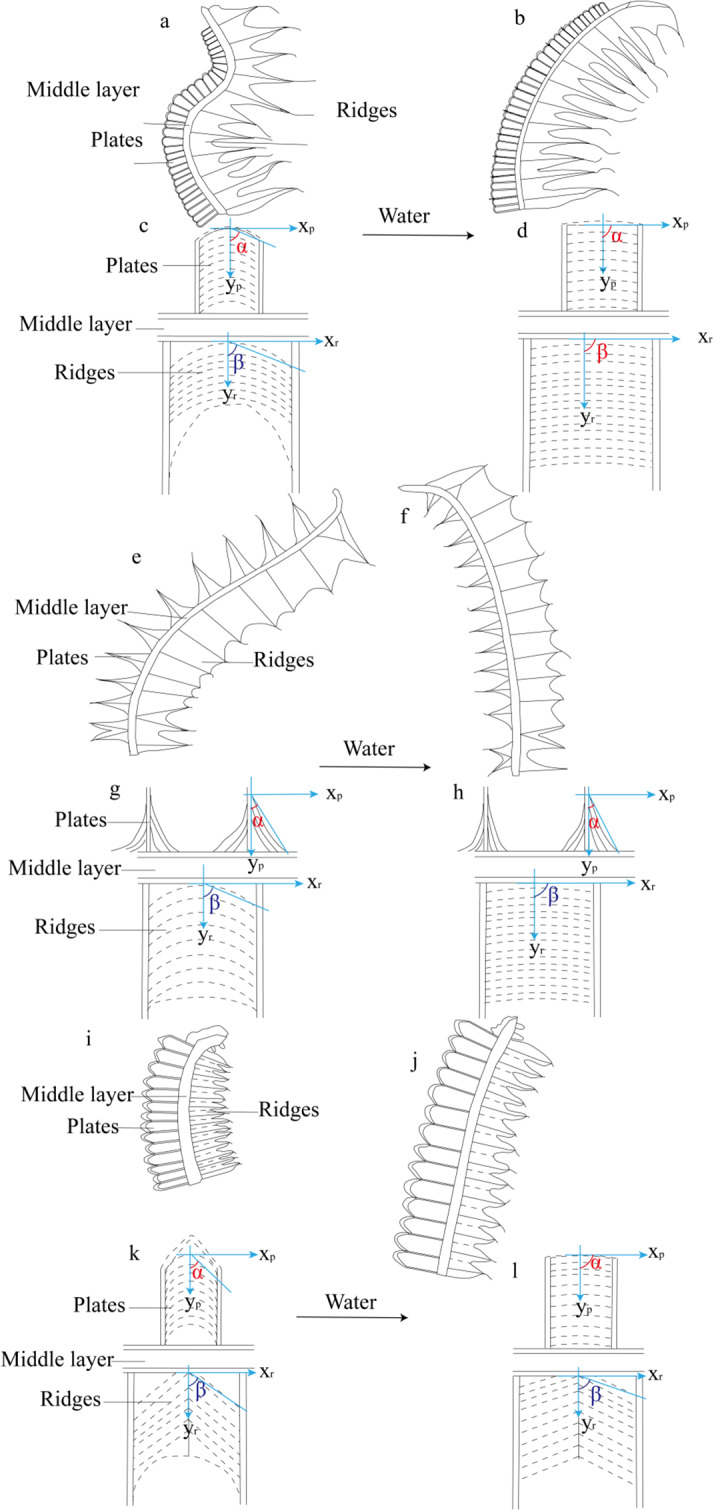
Wetting motion state of the three moss exostomes and microfibrils arrangement angles of outer plates and inner ridges. **a-d**: The exostomes of *H. fauriei*; **a**: The dry state, b. The wet state, **c**: Schematic diagram of microfibrils in dry state (dashed line indicates fine microfibrils, and coarse microfibrils; **α**: the Angle of the fine microfibrils on the plates; **β**: the angle of coarse microfibrils on ridges), **d**: Schematic diagram of microfibrils in wet state; **e-h**: The exostomes of *P. levieri*; **e**: The dry state, f. The wet state, **g**: Schematic diagram of microfibrils in dry state, **h**: Schematic diagram of microfibrils in wet state; **i-l**: The exostomes of *R. declinatus*; **i**: The dry state, **j**: The wet state, **k**: Schematic diagram of microfibrils in dry state, **l**: Schematic diagram of microfibrils in wet state

Microfibrils are believed to be the primary driver of hygroscopic movement in exostomes. Wetting causes these microfibrils to stretch, while drying triggers a strong shrinking response (Figs. 5 and 6) [19]. Radial microfibrils provide the function of radial force, and axial microfibrils exert an axial force (Fig. 6b, e, h). Our observation of arched fine microfibrils aligns with Schnepf’s description. Additionally, we identified a lighter type of coarse microfibril present in a brighter region previously thought to contain less matrix (Fig. 5, Figure S1, Figure S2) [18]. Notably, both types of microfibrils were found in all six moss classes examined, suggesting their widespread occurrence within this group (Figure S2).

*P. levieri* endostomes exhibit significant hygroscopic movement (Fig. 1c, d), potentially linked to microfibril arrangement (Figure S1b, d, f) and possibly influenced by hygroscopic movement in exostomes or the capsule [23].

The arc-axial microfibrils in the outer plates and the inner ridges of exostomes provide axial force, acting as the main driving force for movement (Fig. 5a, b, d, e, g, and h). The arc-axial microfibrils likely play a major role, and the change in their radian (angle) during dry and wet conditions affects the hygroscopic movement of exostomes (Figs. 5b, c, e, f, h and i and 6) [18]. The axial coarse microfibrils in the middle layer of the exostomes may offer additional support. The coarser and denser the microfibrils, the stronger their hardness (Figure S1a, c, and e), making them more prone to fracture during movement. This explains why the exostomes of some species, like *H. fauriei*, are easily fractured after repeated hygroscopic movement [12]. However, movement can also occur in species with highly simplified lateral structures [9, 31–36]. This suggests that the arrangement of microfibrils directly determines movement (Fig. 4, Figure S1, and Figure S2). Coarse and fine microfibrils may be formed by the deposition of different macromolecular polysaccharides, resulting in varying thicknesses of the fibers [37, 38]. The specific components of these two fibers and the physiological processes of their maturation require further investigation.

## Conclusion

The hygroscopic movements of the exostomes differed among the three mosses studied. Hydration caused inward closure in *H. fauriei*, outward opening in *P. levieri*, and elongation in *R. declinatus*. Assuming minimal fiber sedimentation on the inner ridges, we observed the following patterns: (1) Little to no sedimentation on underlying and middle fibers of the outer plates: The exostomes remained open when wet but closed when dry. (2) Gradual decrease in sedimentation from base to middle fibers: Exostomes exhibited greater movement in the middle portion. (3) Similar sedimentation on base and middle fibers: Movement was primarily confined to the base, with minimal elongation. The axial arcs of the outer plates and inner ridges exert force on the microfibrils on both sides, collectively influencing the hygroscopic movement. Consequently, diversified exostome structures across species lead to distinct types of movement, akin to different “dancing” styles. Additionally, the endostomes of *P. levieri* exhibit hygroscopic movement.

### Electronic supplementary material

Below is the link to the electronic supplementary material.

Supplementary Material 1

## Data Availability

No datasets were generated or analysed during the current study.

## Abbreviations

*H. fauriei*: *Hypopterygium fauriei*
*P. levieri*: *Pylaisia levieri*
*R. declinatus*: *Regmatodon declinatus*
SEM: Scanning electron microscopy
TEM: Transmission electron microscopy
EX: Exostomes
EN: Endostomes

## References

[1] Hedwig J. In: Britannica E, editor. Fundamentum Historiae Naturalis Muscorum frondosorum: concernens eorum flores, fructus, seminalem propagationem adiecta generum dispositione methodica, iconibus illustratis. Lipsiae: Apud Siegfried Lebrecht Crusiu; 1782.

[2] Mitten W. Musci Indiae Orientalis: an enumeration of the mosses of the East Indies: Longman, Brown, Green. Longmans & Roberts, and Williams and Norgate; 1859.

[3] Blomquist HL, Robertson LL. The development of the peristome in *Aulacomnium heterostichum*. Bull Torrey Bot Club. 1941;68(8):569–84. 10.2307/2481457

[4] Evans AW, Hooker HD. Development of the peristome in Ceratodon purpureus. Bull Torrey Bot Club. 1913;40(3):97–109. 10.2307/2479567

[5] Taylor EC. The Philibert peristome articles. An abridged translation. Bryologist. 1962;65(3):175–212. 10.1639/0007-2745(1962)65[175:TPPAAA]2.0.CO;2

[6] Chengyi L, Zhihui W, Zhaohui Z. Advances on the Development, structure and function of Moss Peristome. Acta Bot Boreali-Occidentalia Sinica. 2019;10(39):1892–900.

[7] Shaw J, Anderson LE. Morphology and Homology of the Peristome Teeth in *Hymenodon* and *Hymenodontopsis* (Rhizogoniaceae: Musci). Systematic Botany. 1986;11(13):446 – 54.

[8] Ingold C, editor. Peristome teeth and spore discharge in mosses. Transactions of the Botanical Society of Edinburgh; 1959:Taylor & Francis.

[9] Anderson LE, Palmer PG. The Peristome of Anacamptodon splachnoides. Bryologist. 1982;85(2):193–203. 10.2307/3243002

[10] Derschau Mv. Die Entwickelunig Der Peristoinziihiie Des Laubmoossporogoniums. Bot, editor. Centralbl: Neske Stuttgart; 1900. pp. 161–8.

[11] Ye Z-H, Zhong R. Cell wall biology of the moss *Physcomitrium patens*. J Exp Bot. 2022;73(13):4440–53. 35348679 10.1093/jxb/erac122

[12] Lazarenko A. On some cases of singular behavior of the moss peristome. Bryologist. 1957;60(1):14–7. 10.1639/0007-2745(1957)60[14:OSCOSB]2.0.CO;2

[13] Gallenmüller F, Langer M, Poppinga S, Kassemeyer H-H, Speck T. Spore liberation in mosses revisited. AoB Plants. 2018;10(1):plx075. 29372045 10.1093/aobpla/plx075 PMC5777488

[14] Ma WZ, Shevock JR, He S. The first discovery on the sporophytes of a rheophytic moss: *Bryocrumia vivicolor* (Bryophyta, Hypnaceae). Phytotaxa. 2016;265(1):73–8. 10.11646/phytotaxa.265.1.7

[15] Bansal P, Nath V. Peristome as a potential tool for delimiting *Bryum* Hedw.(Bryaceae) from India. Protoplasma. 2018;255:1267–80. 29484492 10.1007/s00709-018-1226-0

[16] Patterson PM. The aberrant behavior of the peristome teeth of certain mosses. Bryologist. 1953;56(3):157–9. 10.1639/0007-2745(1953)56[157:TABOTP]2.0.CO;2

[17] Steinbrinck C. The hygroscopic mechanism of the moss peristomes. Flora. 1897;84:131–58.

[18] Schnepf E, Stein U, Deichgräber G. Structure, function, and development of the peristome of the moss, *Rhacopilum Tomentosum*, with special reference to the problem of microfibril orientation by microtubules. Protoplasma. 1978;97:221–40. 10.1007/BF01276695

[19] Bruggeman-Nannenga MA. On the peristomes of the corticolous African species of *Fissidens* Hedw.(Fissidentaceae, Bryophyta). Cryptogamie Bryologie. 2022;43(2):9–36. 10.5252/cryptogamie-bryologie2022v43a2

[20] Koponen A. Entomophily in the Splachnaceae. Bot J Linn Soc. 1990;104(1–3):115–27. 10.1111/j.1095-8339.1990.tb02214.x

[21] Wu P, Chinese, Mosses. Volume 6. Wu P, editor Beijing: Science; 2002.

[22] Tomotsugu A. A taxonomic study of the genus *Pylaisia* (Hypnaceae, Musci) [Doctoral dissertation]. Tokyo: The University of Tokyo; 2004.

[23] Wu Y, Wang Z, Zhang Z. Telescopic peristomes, hygroscopic movement, and the spore release model of *Regmatodon Declinatus* (Leskeaceae Bryophyta). AoB Plants. 2023;16(6):1–10. 10.1093/aobpla/plad073 PMC10656297 38028746

[24] Goffinet B, buck WR, Shaw J. Morphology, anatomy, and classification of the Bryophyta. Bryophyte Biology. 2008:55–138.

[25] Vitt DH. Adaptive modes of the moss sporophyte. Bryologist. 1981;84(2):166–86. 10.2307/3242820

[26] Hedenäs L. Environmental factors potentially affecting character states in pleurocarpous mosses. Bryologist. 2001;104(1):72–91. 10.1639/0007-2745(2001)104[0072:EFPACS]2.0.CO;2

[27] Johansson V, Lönnell N, Rannik Ü, Sundberg S, Hylander K. Air humidity thresholds trigger active moss spore release to extend dispersal in space and time. Funct Ecol. 2016;30(7):1196–204. 10.1111/1365-2435.12606

[28] Zanatta F, Vanderpoorten A, Hedenäs L, Johansson V, Patiño J, Lönnell N, et al. Under which humidity conditions are moss spores released? A comparison between species with perfect and specialized peristomes. Ecol Evol. 2018;8(23):11484–91. 30598750 10.1002/ece3.4579 PMC6303758

[29] Zhang ZQ, Min HX, Wang ZH, Zhang ZH. Study on spore release of *Polytrichum commune* hedw. Var. Commune by synergetic effects of sub-hygroscopic movement and wind. Plant Biol. 2021;23(6):1018–26. 33988916 10.1111/plb.13285

[30] Whitaker DL, Edwards J. *Sphagnum* Moss disperses spores with vortex rings. Science. 2010;329(5990):406. 20651145 10.1126/science.1190179

[31] Shaw J. Peristome structure in the Orthotrichaceae. J Hattori Bot Lab. 1986;60:119–36.

[32] Garilleti R, Lara F, Albertos B, Mazimpaka V. Peristomal ornamentation, a precise character for discrimination of *Ulola Bruchii* and *U. Crispa* (Bryopsida, Orthotrichaceae). J Bryology. 2000;22:273–8. 10.1179/jbr.2000.22.4.273

[33] Caparros R, Lara F, Long DG, Mazimpaka V, Garilleti R. Two new species of *Ulota* (Orthotrichaceae, Bryopsida) with multicellular spores, from the Hengduan Mountains, Southwestern China. J Bryology. 2011;33(3):210–20. 10.1179/1743282011Y.0000000008

[34] Fedosov VE, Fedorova AV, Fedosov AE, Ignatov MS. Phylogenetic inference and peristome evolution in haplolepideous mosses, focusing on Pseudoditrichaceae and Ditrichaceae s. l. Bot J Linn Soc. 2016;181(2):139–55. 10.1111/boj.12408

[35] Allen NS, Gudiño JA. *Octoblepharum peristomiruptum* (Octoblepharaceae) a new species from the Neotropics. PhytoKeys. 2020;164:1–9. 10.3897/phytokeys.164.51783 PMC7593322 33173400

[36] Ignatov MS, Splrlna UN, Kolesnlkova MA, Ignatova EA. How opposite may differ from opposite: a lesson from the peristome development in the moss Discelium. Bot J Linn Soc. 2020;195(3):420–36. 10.1093/botlinnean/boaa085

[37] Derba-Maceluch M, Awano T, Takahashi J, Lucenius J, Ratke C, Kontro I, et al. Suppression of xylan endotransglycosylase PtxtXyn10A affects cellulose microfibril angle in secondary wall in aspen wood. New Phytol. 2014;205:666–81. 25307149 10.1111/nph.13099

[38] McFarlane HE, Döring A, Persson S. The Cell Biology of Cellulose Synthesis. Annu Rev Plant Biol. 2014;65:69–94. 24579997 10.1146/annurev-arplant-050213-040240

