# Supplementary material for: Microscopic and submicroscopic exploration of diplolepideae peristome structures in hygroscopic movement
Source: BMC Plant Biol. 2024 Jul 26;24:710. doi: 10.1186/s12870-024-05407-8 (PMC11282784; doi:10.1186/s12870-024-05407-8)
Supplement: Supplementary file 1 — Supplementary Material 1 [file 12870_2024_5407_MOESM1_ESM.docx]

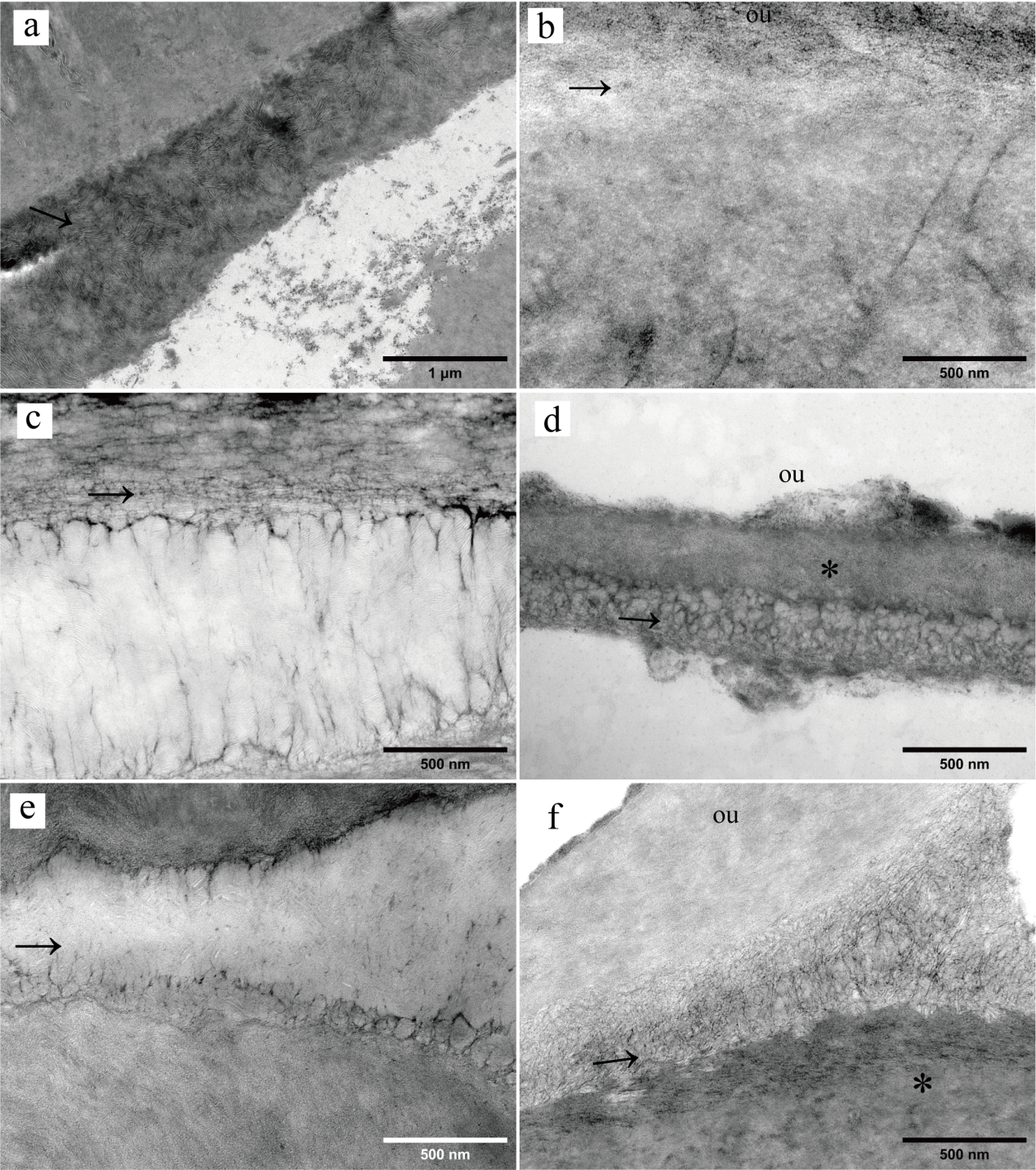


Figure S1 Fiber arrangement of the three mosses exostomes and endostomes (arrows are concentrated distribution areas of coarseness microfibrils in the middle layer; * is a concentrated distribution area of fine microfibril with endostomes; ou stands for dorsal surface). **a-b:** *H. fauriei*; **a:** The middle layer of exostomes, **b:** The endostomes; **c-d:** *P. levieri*; **c:** The middle layer of exostomes, **d:** The endostomes; **e-f:** *R. declinatus*; **e:** The middle layer of exostomes, **f:** The endostomes.


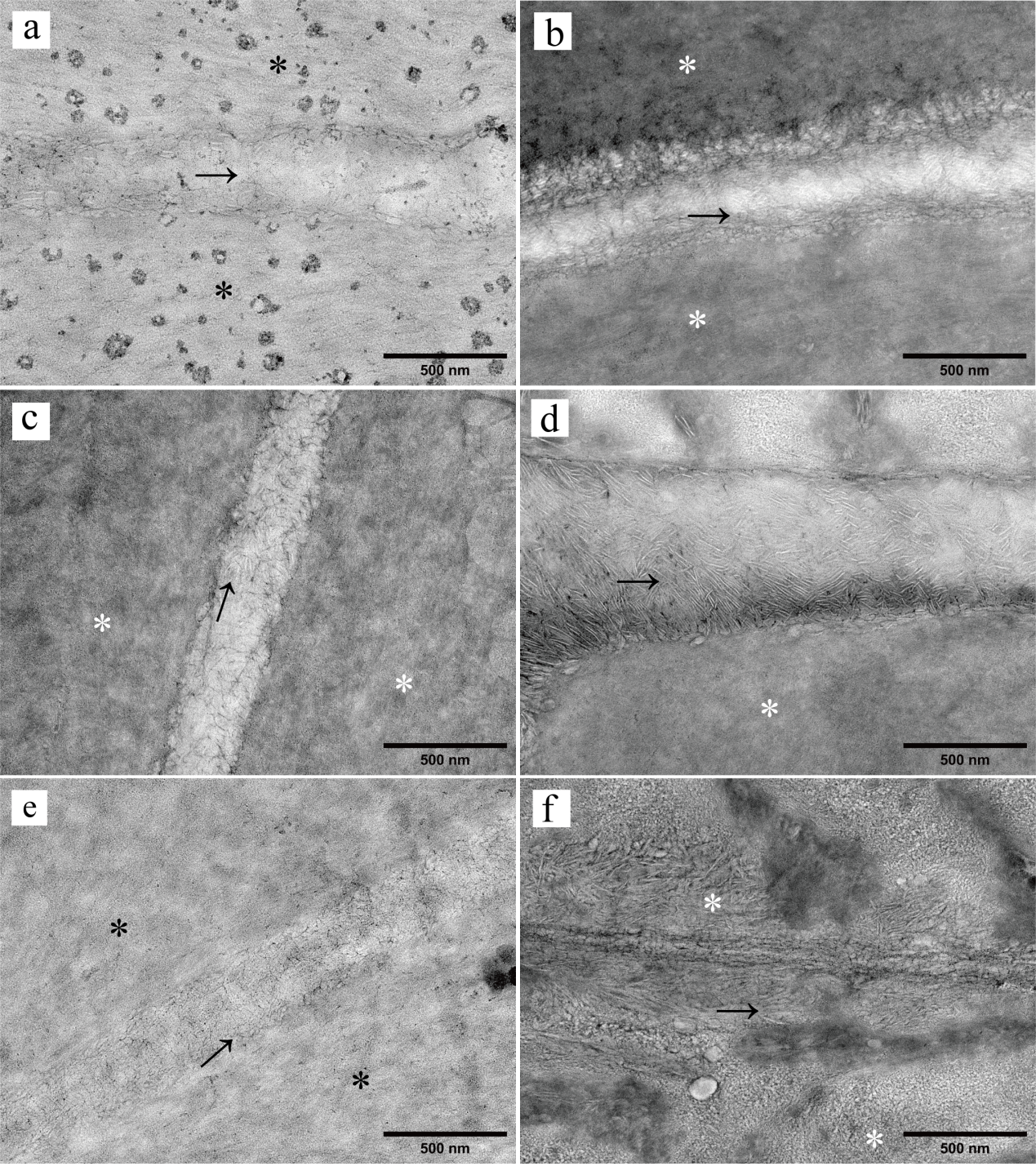


Figure S2 Fiber arrangement of the other six mosses exostomes and endostomes(arrows are concentrated distribution areas of coarse microfibrils in the middle layer; * is a concentrated distribution area of fine microfibril with endostomes; **ou:** dorsal surface). **a:** *Leptocladium sinense* (Broth.); **b:** *Cyrto-hypnum pygmaeum* ((Schimp.) W. R. Buck & H. A. Crum); **c:** *Pseudotaxiphyllum densum* (Cardot) Z. Iwats.）; **d:** *Cyrto-hypnum tamariscellum* (Müll. Hal.) W. R. Buck & Crum）; **e:** *Trichosteleum lutschianum* ((Broth. & Paris) Broth. ); **f:** *Thuidium tamariscinum* ((Hedw.) Schimp. ).
